# Xpert MTB/RIF Ultra Assay Using Stool: an Effective Solution for Bacilli Identification from Adult Pulmonary Tuberculosis Suspects without Expectorated Sputum

**DOI:** 10.1128/spectrum.01265-23

**Published:** 2023-06-28

**Authors:** Xia Yu, Fen Wang, Ruyan Ren, Lingling Dong, Yi Xue, Liping Zhao, Junnan Jia, Hairong Huang

**Affiliations:** a National Clinical Laboratory on Tuberculosis, Beijing Key Laboratory for Drug Resistant Tuberculosis Research, Beijing Chest Hospital, Capital Medical University, Beijing Tuberculosis and Thoracic Tumor Institute, Beijing, Beijing, China; Johns Hopkins University School of Medicine

**Keywords:** pulmonary tuberculosis, Xpert MTB/RIF, MTB Xpert/RIF Ultra, stool

## Abstract

This study evaluated the diagnostic performance of stool-based Xpert MTB/RIF Ultra assay (Xpert-Ultra, Cepheid, USA) against other tests using respiratory tract specimens (RTS) and stool for diagnosing adult pulmonary tuberculosis. A prospective study on patients with presumptive pulmonary tuberculosis was conducted in Beijing Chest Hospital from June to November 2021. The smear test, MGIT960 liquid culture, and Xpert MTB/RIF (Xpert, Cepheid, USA) were performed simultaneously on RTS, and smear, culture Xpert, and Xpert-Ultra were performed simultaneously using stool. Patients were grouped based on the outcomes of RTS examination and other tests. In total, 130 eligible patients were enrolled that included 96 pulmonary tuberculosis and 34 non-TB patients. The sensitivity of smear, culture, Xpert, and Xpert-Ultra using stool was 10.96%, 23.28%, 60.27%, and 79.45%, respectively. The specificities of Xpert and Xpert-Ultra using RTS and stool were all 100% (34/34). Notably, all five confirmed cases detected by bronchoalveolar lavage fluid (BALF) examination yielded Xpert-Ultra positive outcomes with the stool specimens. Xpert-Ultra assay on stool sample harbors comparable sensitivity with Xpert on RTS. Thus, the Xpert-Ultra testing on stool specimens could be a very promising and practical strategy to improve pulmonary tuberculosis (PTB) diagnosis, especially among patients who could not expectorate sputum.

**IMPORTANCE** This study is aimed at assessing the value of Xpert MTB/RIF Ultra (Xpert-Ultra) in PTB on stool in adult in low HIV settings and Xpert-Ultra assay on stool sample harboring comparable sensitivity with Xpert MTB/RIF on respiratory tract specimens. Although the yield in stool samples by Xpert-Ultra is lower than RTS, it may be useful in detecting disease in presumptive TB patients who cannot expectorate sputum and are not open to BALF collection. In addition, Xpert-Ultra with a “trace call” on stool in adult was highly supportive of PTB.

## INTRODUCTION

Worldwide in 2021, an estimated 10.6 million people became ill with tuberculosis (TB) and the number of deaths caused by TB was 1.4 million (1). Early detection plays a key role in improving the outcomes of anti-TB treatment and also reduces the risk of transmission. Smear microscopy is widely used to diagnose TB, especially in high TB-burden countries, despite its notoriously low sensitivity. Culture is considered the gold standard of confirmatory diagnosis of TB due to its high sensitive feature; however, the time-consuming characteristic in culturing of Mycobacterium tuberculosis (MTB) hinders the timely diagnosis. Molecular diagnostics have been developed to reduce diagnostic gaps and delays in treatment. The World Health Organization (WHO) recommends testing all presumptive TB cases with molecular assays, such as the Xpert MTB/RIF (Xpert) and Xpert MTB/RIF Ultra (Xpert-Ultra) (Cepheid Sunnyvale, CA, USA) (2), with the latter being reported to have higher sensitivity than its first-generation counterpart (3).

Until now, the bacteriological diagnosis of pulmonary tuberculosis (PTB) in adults has mainly relied on sputum examination with smear, culture, and PCR-based assays, including Xpert and Xpert-Ultra (4). Therefore, the performances of these assays are highly dependent on the quality and volume of the sputum. Furthermore, many individuals have challenges to expectorate sputum sample, especially young children, the elderly, severely ill patients, people living with HIV, or pregnant women (4). Under such conditions, induced sputum, bronchoalveolar lavage fluid (BALF), and gastric lavage fluid (mainly for children) serve as the alternative specimen types for diagnosis. Sputum induction delivered by an ultrasonic nebulizer takes about 20 min each time and at least two procedures need to be performed (5). Thus, this operation could only be performed in the health care unit, which is not convenient. However, the yields of induced sputum (i.e., 52%) were reported to be slightly higher than spontaneous sputum (49%) (6, 7), albeit previous studies have shown that BALF obtained good yields for molecular testing (49% to 63%) in smear-negative PTB patients. Due to its invasive and expensive features, bronchoscope examination cannot be generalized. Gastrointestinal specimen has been used for PTB diagnosis for more than 100 years since Meunier performed the first test with gastric lavage in children in 1898 (8). Although commonly used in children, adults are seldom tested with this specimen type due to unknown reason.

Stool specimen is easy to obtain and most importantly does not need any invasive operation (9–13). Several studies have assessed the performance of Xpert-Ultra in children with stool specimens (10–12). Sun et al. showed that the sensitivity of Xpert-Ultra on stool (60.3%, 85/141) and on gastric acid (52.5%, 74/141) were comparable (*P = *0.187) (10). Kabir et al. also demonstrated that Xpert-Ultra on stool specimens could identify 83.3% of the bacteriologically confirmed pediatric PTB, whereas Xpert-Ultra on induced sputum only detected 38.9% of the cases (12). Few studies have evaluated the performance of Xpert-Ultra on stool specimens in adult. One study tested eight stool samples with the sensitivity of 80% (4/5) using culture as the reference standard (14). A recent study performed on stool (*n* = 100) in a high-HIV setting (35% HIV positive cases) showed that the sensitivity and specificity of Xpert-Ultra was 90% (95% CI = 79 to 98) and 91% (95% CI = 76 to 98), respectively (15). Until now, no study has been performed to assess Xpert-Ultra performances on adult stool specimen in low-HIV settings. Hence, such evaluation was performed in this study to understand the value of adult stool specimen.

## RESULTS

### Study population.

**FIG 1:**
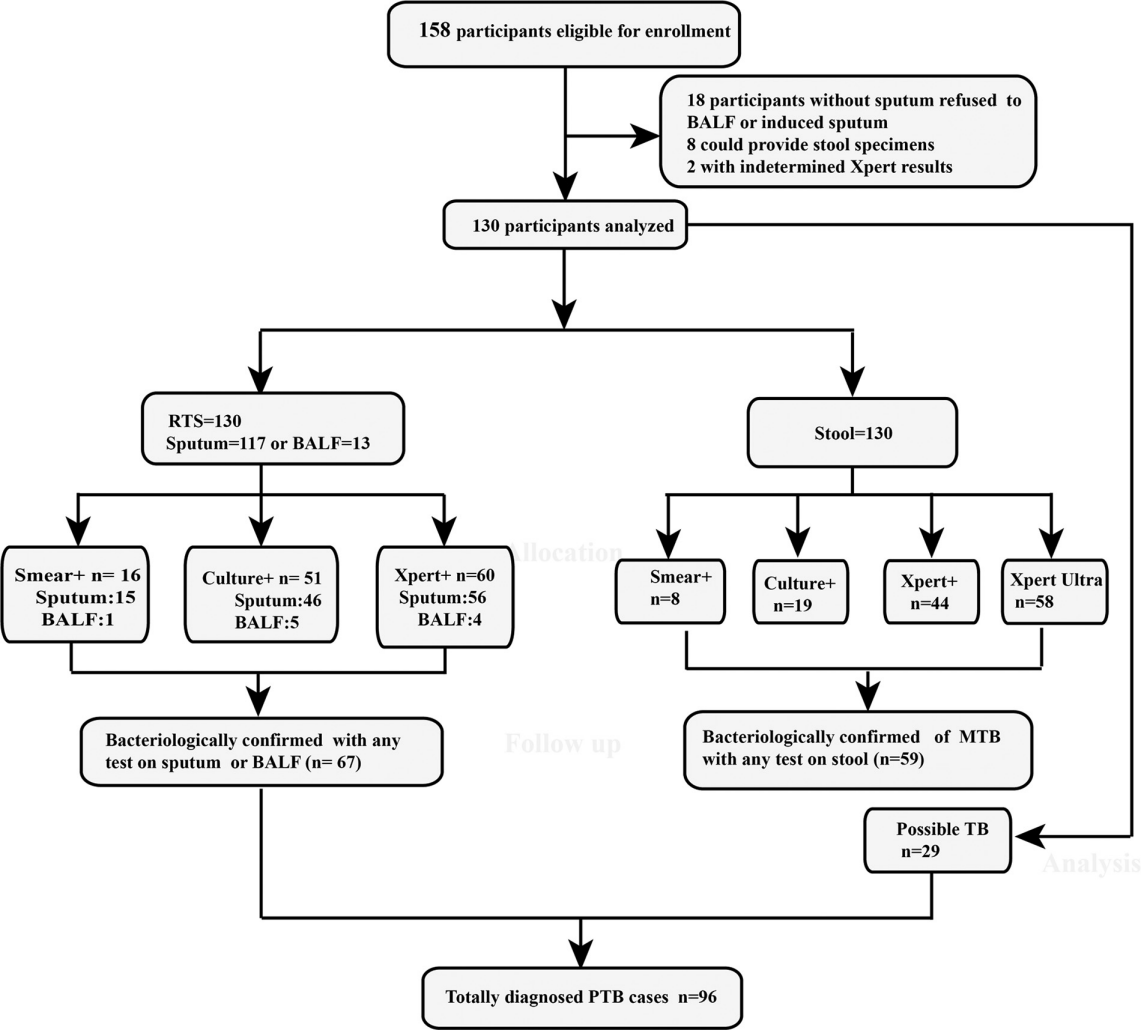
Flowchart depicting the enrollment and investigation results with presumptive pulmonary tuberculosis in adults. BALF, bronchoalveolar lavage fluid; RTS, respiratory tract specimens.

A total of 158 adults with suspected PTB were enrolled, from which 28 cases were excluded, including eight cases that could not provide stool, 18 cases who were unable to expectorate sputum but also refused to undergo bronchoscope examination, one case with invalid Xpert result on sputum, and one case with invalid Xpert-Ultra result on stool. Hence, all the required tests in this study were performed for 130 patients. According to the test on respiratory tract specimens (RTS), 67 patients were diagnosed as confirmed TB whereas 29 cases were diagnosed as probable TB. The other 34 cases were classified as non-TB patients, which included six lung cancer and 28 other infectious disease cases. Two patients yielded positive culture outcomes and were identified as Mycobacterium intracellulare, and were then grouped into non-TB category (Fig. 1). All patients were HIV negative and the demographics and clinical characteristics of all the enrolled cases are shown in Table 1, which demonstrated no statistical difference considering the involved characteristics among the three groups.

**TABLE 1:** Demographic and clinical characteristics of the study participants

Characteristics	TB (*n* = 96)				
	Total (*n* = 96)	Confirmed TB (*n* = 67)	Probably TB (*n* = 29)	Non-TB (*n* = 34)	*P* value
Gender: male/female					0.879
Male	62 (64.6%)	43 (64.2%)	19 (65.5%)	21 (61.8%)	
Female	34 (35.4%)	24 (35.8%)	10 (34.5%)	13 (38.2%)	
Age, avg (range)	53 (18 to 88)	53 (18 to 88)	51 (18 to 81)	54 (22 to 87)	0.913
TB history					0.805
Yes	45 (46.9%)	30 (44.8%)	15 (51.7%)	15 (44.1%)	
No	51 (53.1%)	37 (55.2%)	14 (48.3%)	19 (55.9%)	
On tuberculosis treatment					0.165
Yes	52 (54.2%)	33 (49.3%)	19 (65.5%)	15 (44.1%)	
No	44 (45.8%)	34 (50.7%)	10 (34.5%)	19 (55.9%)	
History of TB contact					0.121
Yes	9 (9.4%)	9 (13.4%)	0 (0%)	2 (5.9%)	
No	87 (90.6%)	58 (86.6%)	29 (100.0%)	32 (94.1%)	
Weight loss					0.898
Yes	35 (36.5%)	25 (37.3%)	10 (34.5%)	13 (38.2%)	
No	61 (63.5%)	42 (62.7%)	19 (65.5%)	21 (61.8%)	
Cough > 2 weeks					0.256
Yes	73 (76.0%)	55 (82.1%)	18 (62.1%)	25 (73.5%)	
No	23 (24.0%)	12 (17.9%)	11 (37.9%)	9 (26.5%)	
Fever					0.718
Yes	34 (35.4%)	24 (35.8%)	10 (34.5%)	14 (41.2%)	
No	62 (64.6%)	43 (64.2%)	19 (65.5%)	20 (58.8%)	

### The performances of different assays in PTB diagnosis.

Considering RTS specimens, 117 cases provide sputa, and 13 cases provided BALF. With sputum, 15 were positive by smear, 46 by culture, and 56 by Xpert. While with BALF, one was positive by smear, five by culture, and four by Xpert (Fig. 1). In total, 67 (51.54%) cases were bacteriologically confirmed on RTS, and 62 and five cases were identified according to the outcomes of sputum or BALF, respectively.

**FIG 2:**
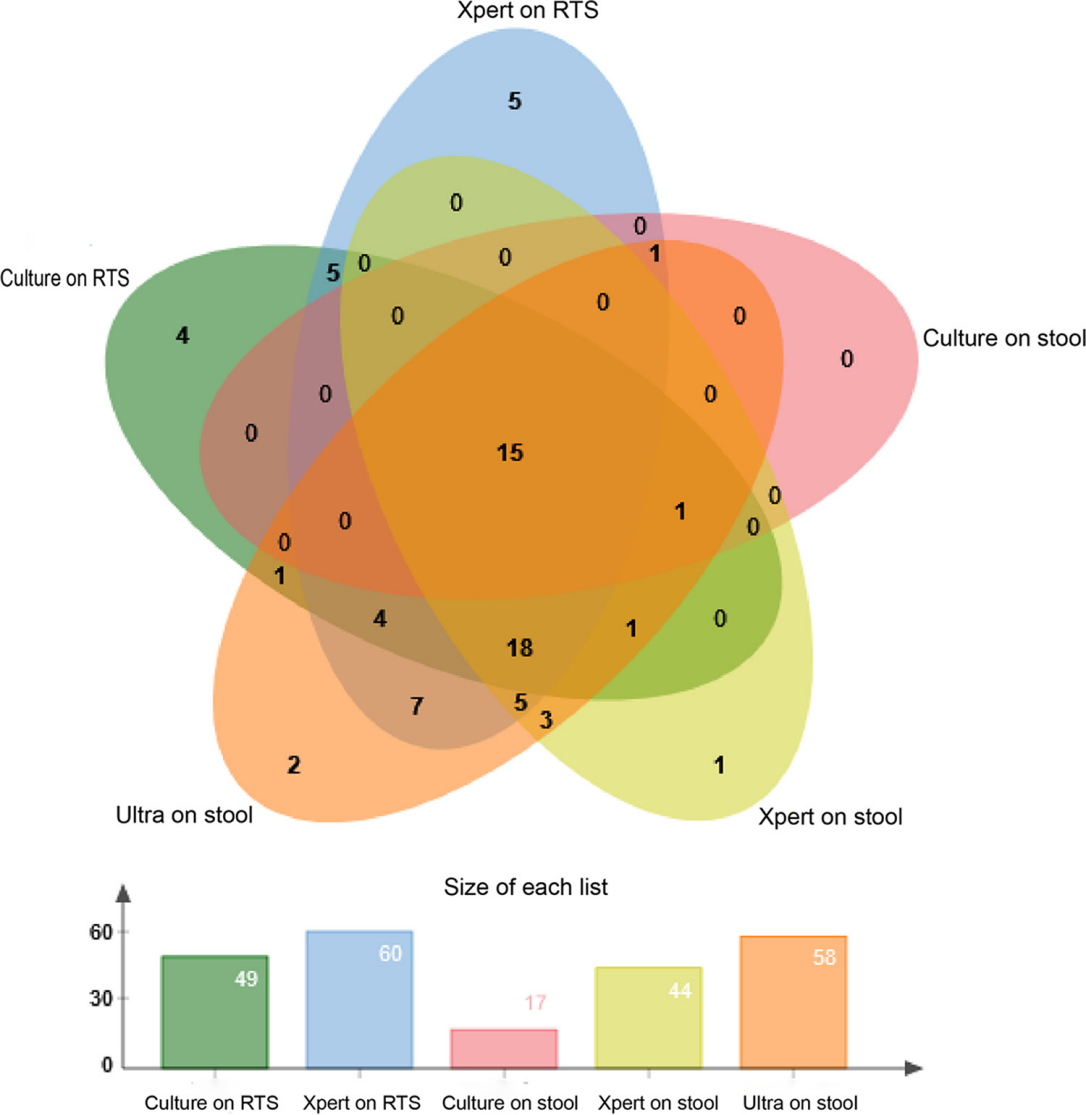
Venn diagram depicting bacteriological confirmation using culture, Xpert MTB/RIF, and Xpert MTB/RIF Ultra assays on RTS and stool specimens with presumptive pulmonary tuberculosis in adults.

Among the 96 PTB patients, 60 (62.50%) were identified by Xpert on RTS, 44 (45.83%) by Xpert on stool, and 58 (60.42%) by Xpert-Ultra on stool. Among the 58 cases positive on Xpert-Ultra, 12 (20.69%) had a trace call (Table 2). The sensitivity and specificity of Xpert-Ultra on stool were 60.42% (95% CI = 49.89 to 70.10) and 100.00% (95% CI = 87.36 to 100.00), respectively. With RTS, Xpert produced comparable sensitivity of 62.50% (95% CI = 51.99 to 72.00) to Xpert-Ultra on stool (Table 3).Notably, four and five of the probable PTB patients, categorized according to the RTS examination, produced positive outcomes by Xpert or Xpert-Ultra on stool specimens, respectively. Therefore, extra examinations with stool added value for confirmed TB diagnosis for Xpert and Xpert-Ultra by 4.17% and 5.21%, respectively. In contrast, this value for culture on stool was zero (Fig. 2). Furthermore, all five confirmed cases detected by BALF examination produced positive outcomes by Xpert-Ultra on stool specimens, while culture and Xpert on stool examination detected two or three of them separately.

**TABLE 2:** Semiquantitative categories of Xpert MTB/RIF and Xpert MTB/RIF Ultra assays with bacteriologically confirmed and probable PTB on RTS and stool specimens

Investigation	BALF group (*n* = 13)			Sputum group (*n* = 117)			Total		
	Xpert-BALF	Xpert-stool	Ultra-stool	Xpert-sputum	Xpert-stool	Ultra-stool	Xpert-RTS	Xpert-stool	Ultra-stool
Total positive	4	4	6	56	40	52	60	44	58
High	0	0	0	7	0	1	7	0	1
Medium	0	0	0	13	6	6	13	6	6
Low	3	0	1	19	14	20	22	14	21
Very low	1	4	2	17	20	16	18	24	18
Trace call	NA^*a*^	NA	3	NA	NA	9	NA	NA	12

a NA, not applicable.

**TABLE 3:** Performance of different methods for pulmonary tuberculosis diagnosis on the different types of specimens using patient category as the golden standard

Diagnostics	Specimen type	Confirm TB *n* = 67	Possible TB *n* = 29	Non-TB *n* = 34	Sensitivity % (95% CI)	Specificity % (95% CI)	PPV % (95% CI)	NPV % (95% CI)
Xpert-Ultra	Stool	53/67	5/29	0	60.42 (49.89, 70.10)	100.00 (87.36, 100.00)	100 (92.26, 100.00)	47.22 (35.48, 59.27)
Xpert	Stool	40/67	4/29	0	45.83 (35.73, 56.28)	100.00 (87.36, 100.00)	100.00 (90.00, 100)	39.53 (29.33, 50.68)
Xpert	RTS	60/67	0/29	0	62.50 (51.99, 72.00)	100.00 (87.36, 100.00)	100.00 (92.50, 100.00)	48.57 (36.57, 60.73)
Culture^*a*^	Stool	27/67	0/29	2	28.13 (19.65, 38.37)	94.11 (78.94, 98.97)	93.10 (75.78, 98.79)	31.68 (22.98, 41.79)
Culture^*a*^	RTS	49/67	0/29	2	51.04 (40.69, 61.31)	94.12 (78.94, 98.97)	96.08 (85.41, 99.32)	40.51 (29.79, 52.15)

a Two patient with sputum and stool culture positive was identified to be infected by M. intracellulare and classified to non-TB group.

Using RTS culture as the reference test, the sensitivity of Xpert on stool was 68.63% (95% CI = 53.97 to 80.48) and that of Xpert-Ultra was 78.43% (95% CI = 64.30 to 88.25). The specificity was 88.61% (95% CI = 78.99 to 94.34) for Xpert and 77.22% (95% CI = 66.15 to 85.59) for Xpert-Ultra (Table 4).

**TABLE 4:** Diagnostic accuracy of Xpert MTB/RIF and Xpert MTB/RIF Ultra assays on stool specimen compared with culture on RTS specimen with presumptive pulmonary tuberculosis

Diagnostics	Result	Culture on RTS^*a*^					
		Positive (*n* = 51)		Negative (*n* = 79)			
		No.	%	No.	%	Sensitivity	Specificity
Xpert MTB/RIF on RTS	Positive	42	82.35	18	22.78	82.35 (68.64 to 91.13)	77.22 (66.15 to 85.59)
	Negative	9	17.65	61	77.22		
Xpert MTB/RIF on stool	Positive	35	68.63	9	11.39	68.63 (53.97 to 80.48)	88.61 (78.99 to 94.34)
	Negative	16	31.37	70	88.61		
Xpert MTB/RIF Ultra on stool	Positive	40	78.43	18	22.78	78.43 (64.30 to 88.25)	77.22 (66.15 to 85.59)
	Negative	11	21.57	61	77.22		

a RTS, respiratory tract specimens.

Among the 32 non-TB patients, two cases had culture-positive outcome on both sputa and stool examination, and the isolates were subsequently identified as M. intracellulare. All other tests on sputum and stool were negative in this patient group. Therefore, the specificities of smear, Xpert, and Xpert-Ultra on RTS and stool were all 100% (32/32), while for MGIT960 liquid culture it was 94.12% (32/34) for RTS and stool.

### Diagnostic yields using RTS and stool specimens.

Of the 67 bacteriologically confirmed PTB cases, Xpert on RTS presented the diagnostic yield of 60 cases (89.55%), while Xpert Ultra on stool specimen detected 58 cases (86.57%) as positive (Table 5). Culture on stool showed the lower diagnostic yield with 17 cases in 96 diagnosed TB cases; the diagnostic yield of Xpert Ultra does not increase (60.42%) when integrating with it. Of the 96 total diagnosed TB cases, Xpert Ultra on stool specimen showed the clarify detection of 58 cases, which is comparable with the highest diagnostic yield of 67 cases by culture plus Xpert on RTS (χ^2^ = 1.857, *P* = 0.173) (Table 5).

**TABLE 5:** Diagnostic yield of different laboratory assays on RTS and stool specimens among pulmonary tuberculosis

Investigations	No. of positive	Bacteriologically confirmed TB (%) (*n* = 67)	Total diagnosed TB^*a*^ (%) (*n* = 96)
Only smear on RTS	16	23.88 (16/67)	16.67 (16/96)
Only culture on RTS	49	73.13 (49/67)	51.04 (49/96)
Only Xpert on RTS	60	89.55 (60/67)	62.50 (60/96)
Culture plus Xpert on RTS	67	100 (67/67)	69.79 (67/96)
Only smear on stool	8	11.94 (8/67)	8.33 (8/96)
Only culture on stool	17	25.37 (17/67)	17.71 (17/96)
Only Xpert on stool specimen	44	65.67 (44/67)	45.83 (44/96)
Only Xpert Ultra on stool specimen	58	86.57 (58/67)	60.42 (58/96)
Culture plus Xpert on stool	45	67.16 (45/67)	46.88 (45/96)
Culture plus Xpert Ultra on stool	58	86.57 (58/67)	60.42 (58/96)

a Total diagnosed TB, bacteriologically confirmed TB plus probable TB cases.

## DISCUSSION

Many presumptive adult PTB individuals fail to expectorate sputum; therefore, an alternative, non-sputum-based sample for PTB diagnosis is necessary (16). In recent years, the advantages of stool specimens have been noticed mainly in children (17). Favorable yields had been acquired in Xpert assay with this noninvasive and easy-to-be-obtained specimen. A recent study manifested that adult PTB patients had high acid-fast bacilli (AFB) loads in stool specimens with an overall mean of 5.1 ± 1.59 log_10_ CFU per mL, which strongly supports the usage of stool specimen in PTB diagnosis (15). Serial studies demonstrated that Xpert-Ultra had a high detection rate using stool in pediatric TB and could be used as an alternative specimen of gastric acid. However, perhaps because adults are generally abhorrent to this specimen type, stool specimen is less frequently used for AFB detection. Therefore, the performances of conventional tests and molecular diagnostics on stool specimens have not been fully evaluated in contrast with other specimen types. To the best of our knowledge, our study is the first to evaluate the diagnostic accuracy of Xpert-Ultra on stool specimens for PTB diagnosis in adults in low HIV setting. Notably, our study found that the performance of Xpert-Ultra on stool specimens was comparable to that of Xpert on RTS, which supported its usage as an alternate, noninvasive specimen type for PTB diagnosis in adults, especially for patients who are unable to produce a sputum specimen.

Stool samples have recently been recommended by WHO as a specimen type of molecular TB diagnosis in children, whereas a well-recognized processing method of stool specimen is not available (18). Two recent meta-analyses showed that the performances of Xpert assay on stool varied greatly between studies, with sensitivity varying between 25% and 85% across studies, while the different processing methods used in different studies were considered the major cause of variation (13, 19). Previous studies demonstrated that centrifuge-free stool processing was easy and could acquire high yields with the lowest invalid error (4.5%). According to these studies, the best practice includes using 0.5 g stool, manual shaking, 30-min sedimentation, and processing with a 1:3.6 dilution ratio by Xpert sample processing reagent (20). In our study, we followed the recommended procedure of sputum from the manufacturer with 1 g stool, and only one out of the 130 stool specimens reported invalid results (0.77%, 1/130). The stool processing method we used is rapid and simple and could avoid the loss of bacilli caused by centrifugation steps. Until now, there is no acceptable standardized technique for stool processing of Xpert-Ultra. Therefore, our experience would give some insights into establishing a standard processing method of stool specimen for the Xpert-Ultra assay.

Overall, for a given test, the stool test had less favorable detection rate compared with sputum directly, especially for culture. MGIT960 liquid culture on stool had low diagnostic accuracy in this study, whether in bacteriologically confirmed cases (25.37%, 17/67) or in total diagnosed PTB cases (17.71%, 17/96). These outcomes were consistent with the findings from other studies which showed that molecular assays had a higher positive rate than culture on stool (15, 21). The low detection rate of culture on stool may partly be attributed to loss of viability during the transition in the stomach and gut, whereas this harsh condition was less influential on molecular tests which targeted the DNA of MTB. Thus, molecular testing is more preferable over culture on stool for TB diagnosis.

In principle, MTB or its DNA detected in stool was originally from swallowed excretion of the respiratory tract. Therefore, it was not surprising to see that the stool had relatively inferior detection efficiency than sputum. In general, the added value with these extra tests on stool specimens was weak. In total, only six extra cases acquired bacteriological evidence with stool examination among the probable PTB patients, which incurred an 8.22% increase in the confirmed PTB cases. Nevertheless, the detection capacity of Xpert-Ultra on stool (60.42%, 58/96) was comparable to the Xpert assay on sputum (62.50%, 60/96), which strongly supports the usage of Xpert-Ultra on stool specimens in patients whose sputum production is problematic. Notably, all five cases that produced positive Xpert outcomes on BALF were also Xpert-Ultra positive on stool specimens. To some extent, this outcome endorses Xpert-Ultra on stool specimens as a promising strategy to substitute the very invasive bronchoscope examination for AFB detection purposes.

A high proportion of “trace call” positive (20.69%,12/58) was obtained on stool examination in TB patients in our study. In contrast, only seven patients yielded Xpert-Ultra “trace call” outcome on RTS. However, none of the 34 non-TB cases acquired any positive outcome by Xpert or Xpert-Ultra. Overall, treating the Xpert-Ultra trace result on stool as true positive improved the detection sensitivity to a significant scale without any noticeable loss in specificity. Therefore, we suggest taking a “trace call” of Xpert-Ultra on the stool in adults as a positive result for diagnosis of PTB.

There are some limitations in our study. First, this study was performed in a single center with a small sample size. Therefore, the variance in patient constitution might affect the overall performance parameters; larger multicenter studies will need to extend the evidence for this approach. Additionally, in view of the high prevalence of TB in this study site, the specificity and positive predictive value of Xpert or Xpert-Ultra on stool acquired in this study might be overestimated. Second, because there is no recommended processing method for stool specimens, we used the simple one-step (SOS) method. Different processing methods can cause big variance. Third, Xpert-Ultra was not performed on RTS in this study. However, ours and other previous studies reported about a 3% to 5% sensitivity increase on sputum examination with this new generation Xpert assay (22, 23).

In conclusion, the yield in stool samples by Xpert-Ultra is lower than RTS but may be useful in detecting disease in presumptive TB patients who cannot expectorate sputum and are not open to BALF collection.

## MATERIALS AND METHODS

### Ethical approval.

This study was approved by the ethics committee of Beijing Chest Hospital, Capital Medical University. Written informed consents was obtained from all the participants.

### Patient enrollment.

Patients with typical TB symptoms, including cough for more than 2 weeks, fever, and weight loss, as well as with suggestive chest radiography, were recruited prospectively and consecutively in Beijing Chest Hospital from June to November 2021. Eligible participants were at least 18 years old.

### Patient category.

Patients were assigned to one of the following three diagnostic categories according to the outcomes of different laboratory testing with respiratory tract specimens (RTS) and clinical features: (i) bacteriologically confirmed TB: any positive outcome with smear, culture, or Xpert, whereas patients identified with non-tuberculous mycobacteria were excluded and registered in the “non-TB” group; (ii) probable TB: none of the smear, culture, or Xpert was positive AND other pulmonary diseases (including pneumonia, lung tumor, lung cyst, and interstitial lung diseases) were excluded AND good clinical response to anti-TB chemotherapy was observed; and (iii) “non-TB”: other pulmonary diseases were diagnosed according to different laboratory testing and pathological examination.

Patient category was used as the golden standard in our study; bacteriologically confirmed TB and probable TB group were all considered total diagnosed TB group.

### Study procedures and specimen collection.

A prospective cohort study was conducted to evaluate the performances of smear, culture, Xpert, and Xpert-Ultra on the stool for PTB diagnosis in adults at Beijing Chest Hospital from June to November 2021. Sputum was collected and subject to smear, culture, and Xpert assay. For patients who could not produce sputum, BALF was collected as a substitute. Meanwhile, a stool specimen was collected from each enrolled patient and subjected to smear, culture, Xpert, and Xpert-Ultra simultaneously.

### Smear and Mycobacterium culture.

Smear microscopy on RTS (sputum or BALF) with auramine-O staining (Baso, Guanzhou, GuangDong, China) was performed by following mycobacteriology laboratory manual and examined by light-emitting diode microscopy (24). The stool or RTS specimens were processed with a final N-acetyl-cysteine-NaOH of 1% for decontamination (20 min) and digestion before inoculation into the growth vial for MGIT960 liquid culture (BD Diagnostic Systems, NJ, USA) following the manufacturer’s instructions.

### Xpert assay on RTS specimens.

Xpert testing was performed as per the manufacturer’s instructions using a 2:1 ratio of Xpert reagent to the sample. One milliliter of RTS was used. The cartridges were applied to the Xpert platform for detection.

### Xpert and Xpert-ultra detection on stool.

The stool processing method was modified from a SOS stool method (i.e., only including one release/sedimentation step) by Klinkenberg (25). Approximately, a portion of 0.8 to 1.0 g stool with was collected into a 50 mL plastic tube and 2 volumes of Xpert/Xpert-Ultra regent (3 mL) were added to the sample as per the manufacturer’s instructions. The tube was shaken for 30 s, left at room temperature for 5 min, shaken vigorously again for 30 s, and left at room temperature for another 10 min for cell debris to settle by gravity sedimentation, and 2 mL of the top layer liquid was transferred to the Xpert/Xpert-Ultra cartridge. The sample was tested generally on the same day of collection. If sample was not tested on the same day, it was frozen at −80°C until usage.

### Data analysis.

SPSS version 20 was used for the data analysis. Proportions were used to summarize the sociodemographic details, TB history, and symptom profile. Venn diagram depicted the bacteriological confirmation using culture, Xpert, and Xpert-Ultra on RTS and stool specimens. The sensitivity, specificity, positive predictive values (PPV), and negative predictive values (NPV) of the culture, Xpert, and Xpert-Ultra assay were calculated against the reference standard from the following URL: http://vassarstats.net. We also calculated the sensitivity and specificity of Xpert-Ultra for stool specimens by considering trace call as negative.
